# Improved Surface Functional and Photocatalytic Properties of Hybrid ZnO-MoS_2_-Deposited Membrane for Photocatalysis-Assisted Dye Filtration

**DOI:** 10.3390/membranes10050106

**Published:** 2020-05-21

**Authors:** Saranya Rameshkumar, Rory Henderson, Ramesh Babu Padamati

**Affiliations:** 1 AMBER Centre, CRANN Institute, Trinity College Dublin, Dublin 2, Ireland; rameshks@tcd.ie; 2 BiOrbic—Bioeconomy SFI Research Centre, University College Dublin, Belfield, Dublin 4, Ireland; 3 School of Physics, Trinity College Dublin, Dublin 2, Ireland; 4 School of Chemistry, Trinity College Dublin, Dublin 2, Ireland; rhenders@tcd.ie

**Keywords:** photocatalytic membrane, hybrid ZnO-MoS_2_ layer, surfactant-assisted exfoliation, surface modification, membrane filtration, dye degradation

## Abstract

The synergistic mechanism of photocatalytic-assisted dye degradation has been demonstrated using a hybrid ZnO-MoS_2_-deposited photocatalytic membrane (PCM). Few layers of MoS_2_ sheets were produced using the facile and efficient surfactant-assisted liquid-phase exfoliation method. In this process, hydrophilic moieties of an anionic surfactant were adsorbed on the surface of MoS_2_, which aided exfoliation and promoted a stable dispersion due to the higher negative zeta potential of the exfoliated MoS_2_ sheets. Further, the decoration of ZnO on the exfoliated MoS_2_ sheets offered a bandgap energy reduction to about 2.77 eV, thus achieving an 87.12% degradation of methylene blue (MB) dye within 15 min of near UV-A irradiation (365 nm), as compared with pristine ZnO achieving only 56.89%. The photocatalysis-enhanced membrane filtration studies on the ZnO-MoS_2_ PCM showed a complete removal of MB dye (~99.95%). The UV-assisted dye degradation on the ZnO-MoS_2_ PCM offered a reduced membrane resistance, with the permeate flux gradually improving with the increase in the UV-irradiation time. The regeneration of the active ZnO-MoS_2_ layer also proved to be quite efficient with no compromise in the dye removal efficiency.

## 1. Introduction

The membrane filtration process is one of the most versatile and effective means of water/wastewater treatment technologies, owing to its reduced energy consumption and operating costs. The sustainable design of membrane processes is recently gaining more attention, especially with a focus to overcome the fundamental limitations on reject disposal associated with any membrane-based treatment [1]. Indeed, there are several evidences on integrating different unit operations or chemical processes with membrane separation, yet reject disposal remains challenging in terms of the economic and energy aspects [2,3]. Photocatalysis is one of the promising processes to be integrated with membrane separation for its advantage of the incomplete decomposition of organic pollutants under UV/visible light irradiation. Novel process designs in photocatalytic membrane reactors are continuously emerging to realise the maximum benefit from both photocatalysis and membrane filtration processes [4]. However, there are associated limitations with slurry/submerged or continuous photocatalytic membrane reactors in terms of photocatalyst regeneration, poor photocatalytic activity, excessive membrane resistance [5] and fouling [6,7]. Photocatalytic membranes (PCM) enable to overcome the above-mentioned limitations wherein the active photocatalytic layer is immobilised on the surface of the membrane rather than the bulk incorporation [8]. 

As an attempt to utilise the maximum photocatalytic efficiency, the membrane surface can be inherently tailored with desired multi-functionalities. By identifying a suitable material constituting photocatalytic, anti-fouling and selective separation properties, a hybrid PCM with a well-controlled structure and high performance could be developed. The recent advent of high-performance two-dimensional (2D) materials has been a significant impact on developing robust photocatalytic membrane materials [9,10], thanks to the advancements in material chemistry. Several research studies highlighted the photocatalytic activity of novel transition metal chalcogenides (TMDs) and 2D nanomaterials-based semiconductors [11,12,13]. However, studies related to developing membranes with photocatalytically active TMD-based semiconductor nanomaterials are limited. 

The present work is central to achieving the desired surface functionality of the polymeric membranes by incorporating an active TMD layer with a photocatalytic property to perform a UV-assisted membrane filtration process. Special interest also lies in introducing heterojunctions between photocatalytic semiconductors and exfoliated 2D materials to realise a photocatalytic-enhanced membrane treatment. Emerging 2D TMDs, like MoS_2_, typically prepared based on liquid exfoliation [14], or particularly using solvents [15,16] and surfactants [17], hold greater potential in terms of nano-structural stability and permeation capability higher than that of its counterparts like graphene oxide, and has also been evidenced to offer membrane integrity towards pressure-driven water filtration applications [18,19]. As the present work necessitates the incorporation of exfoliated layers on MoS_2_, a facile and environmentally friendly surfactant-assisted exfoliation method [20] has been adopted to prevent any damage of the membrane substrate against solvent exposure and at the same time improve the stability of exfoliated MoS_2_ sheets. MoS_2_ can be effectively integrated as an active layer on the surface of the membrane by taking advantage of the robust van der Waals structure with a stable/narrow interlayer of MoS_2_ in an aqueous environment [21]. Robust surface modification strategies to beneficially utilize the multi-functional properties of MoS_2_ without altering the desired molecular channels within its interlayer spacing would hence bring breakthrough advances in the field of photocatalytic membranes.

Hybrid photocatalytic membranes constituting a selective and photocatalytic surface layer tend to offer synergistic benefits as well as mitigate the trade-off existing between the productivity and rejection efficiency of the membrane. Improved filtration properties and membrane life will be rendered while reducing the typical limitations of membrane filtration concerning reject disposal and fouling. The present study deals with developing a photocatalytically active polyvinylidene difluoride (PVDF) polymeric nanocomposite membrane with few layers of 2D hybrid ZnO-MoS_2_ deposited on its surface. Methylene blue (MB) dye was chosen as a model pollutant to investigate the efficiency of the photocatalytic-assisted membrane filtration performance of a pristine ZnO-MoS_2_ powder and hybrid ZnO-MoS_2_ PCM, respectively. Chemically exfoliated MoS_2_ (E- MoS_2_), having a shorter conduction band gap energy, activates the electron transfer rate of the ZnO semiconductor by suppressing the faster recombination of its electron–hole pairs [1,22] which enable enhanced membrane properties such as surface adsorption, hydrophilic properties and membrane filtration performance [23,24].

## 2. Materials and Methods 

### 2.1. Materials 

Bulk MoS_2_ powders (mesh size <2 microns), sodium dodecylbenzene sulfonate (SDBS) and methylene blue (MB) were purchased from Sigma Aldrich, Dublin, Ireland. N-methyl pyrrolidone (NMP) was purchased from Fisher Scientific Limited, Loughborough, UK. PVDF membrane (pore size ~0.2 microns) substrate was procured from Sterlitech Corporation, Kent, WA , USA. Zinc oxide (ZnO) nanopowders were obtained from Nanostructured & Amorphous Materials, Inc., Katy, TX, USA. All chemicals used were of analytical grade. Deionised water was produced in the laboratory using a Vision 250 deioniser, Veolia Ireland, Dublin, Ireland.

### 2.2. Surfactant Assisted Exfoliation of Bulk MoS_2_

**Figure 1:**
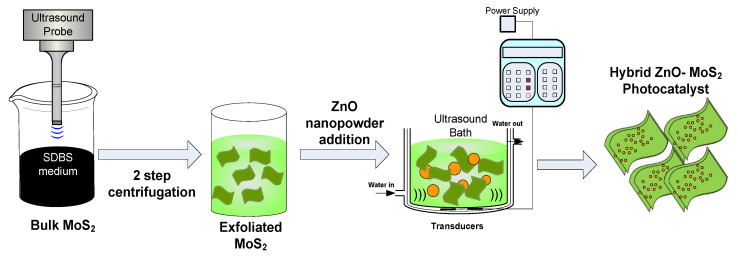
Schematic illustration of E-MoS_2_ and hybrid ZnO-MoS_2_ photocatalyst.

Bulk MoS_2_ (50 mg) was added to a solution of deionised water (50 mL) and SDBS (25 mg). The solution was magnetically stirred while being heated to 45 °C for 20 min. The mixture was then ultra-sonicated for 3 h using a solid probe sonicator (Sonics, Newtown, CT, USA) with a 750W power output set at a 20 kHz frequency and maximum amplitude of 25%. In order to control the temperature, sonication was performed in a jacketed beaker with water circulation and the sonicator was set at a pulse rate of 7 s on and 2 s off [20,25]. The resultant dispersion containing the exfoliated (E-MoS_2_) and bulk unexfoliated (B-MoS_2_) was separated by two-step centrifugation. Initially, the dispersion was centrifuged at 4000 rpm for 1 h, and an upper green liquid part of the dispersion containing the exfoliated layers of MoS_2_ was collected while leaving the larger flakes’ sedimented residue for disposal. The collected supernatant was then centrifuged again for 30 min at 4000 rpm to avoid further bulk residues sedimented at the bottom of centrifuge tubes. Finally, the as-obtained E-MoS_2_ dispersion was used for the membrane modification and further characterisation, as shown in Figure 1.

### 2.3. Preparation of Hybrid ZnO-MoS_2_ Nanocomposite

Twenty mg of ZnO nanopowder was taken per 10 ml of the E-MoS_21_ dispersion (1 mg/mL concentration) and stirred for 20 min. Sonochemical-assisted doping of E-MoS_2_ was carried out by ultra-sonicating the entire solution for 30 min. A greenish white turbid dispersion was obtained, which was stirred further for 2 h [26]. The resultant dispersion was dried in a vacuum oven at 70 °C for 12 h, followed by heating for 2 h at 200 °C to collect the dried ZnO-MoS_2_ nanocomposite [27,28]. The as-obtained hybrid ZnO-MoS_2_ (Figure 1) was characterised and employed in pristine form to study its photocatalytic activity. 

### 2.4. Characterisation of E-MoS_2_ Dispersion and MoS_2_ Doped ZnO

The stability of E-MoS_2_, ZnO and the hybrid ZnO-MoS_2_ nanocomposite was compared by measuring its zeta potential using a zetasizer nano ZS (Malvern Panalytical Ltd.,Worcestershire, UK). ZnO and the as-prepared dried samples of the hybrid ZnO-MoS_2_ were prepared using deionised water (pH 7.6) at a concentration of 3 mg/mL, and the dispersion of E-MoS_2_ was centrifuged and later re-dispersed in deionised water at a similar concentration. All measurements were performed in triplicates to determine the average zeta potential. Raman spectra of E-MoS_2_ and the hybrid ZnO-MoS_2_ photocatalyst were collected by means of a non-resonant 532 nm cw laser. The full-spectrum was recorded while placing the samples on a standard XY motorised stage and a coupled microscope objective (20× with 3 μm laser spot size) helped in scanning and collecting the back-scattered light using a grating spectrometer. Optical analysis was performed using UV–vis spectroscopy (Perkin-Elmer lambda 1050 spectrophotometer, Waltham, MA, USA) to measure the band gap energy of the prepared photocatalyst by means of the well-known Tauc relation, Equation (1).

*h*ν = *A* (*h*ν − *E_g_*)^*n*^ (1)

where *h* refers to the Plank constant, ν is the incident of light frequency, *A* is the absorbance, *E_g_* is the bandgap and *n* equals to ½, which refers to the direct band gap energy of the E-MoS_2_ dispersion. 

### 2.5. Modification of PVDF Membrane with Hybrid ZnO-MoS_2_ Photocatalysts

For the surface modification, 20 mL of the prepared dispersion of the hybrid ZnO-MoS_2_ (3 mg/mL concentration) was deposited on a PVDF membrane with the help of a pre-assembled filtration set-up (Sartorius Filtration apparatus, Microsart®, Göttingen, Germany) operated by a peristaltic pump. Deposition of the synthesised ZnO-MoS_2_ dispersion was confirmed by visualising the clear permeate through the membrane. Finally, the ZnO-MoS_2_ PCM was rinsed with deionised water, dried at 85 °C and immersed in deionised water for further use. 

### 2.6. Characterisation of ZnO-MoS_2_ PCM

#### 2.6.1. Hydrophilicity Characteristics

The contact angles of the virgin PVDF and modified ZnO-MoS_2_ PCM were measured using an assembled goniometer set-up (First Ten Angstroms, FTÅ12, Cambridge, UK) to study its hydrophilicity characteristics. The static sessile drop method was adopted wherein a water droplet of 10 µl was placed on the membrane surface using a Gilmount syringe to measure the equilibrium water contact angle. The average of five readings of the equilibrium water contact angles was then calculated and reported as the mean contact angle of the prepared membranes.

#### 2.6.2. Morphological Characteristics

The morphologies of the hybrid ZnO-MoS_2_ photocatalyst and modified membranes were examined using a field emission scanning electron microscope (Zeiss Ultra plus, Carl Zeiss, Oberkochen, Germany). The membrane was initially pre-treated with gold (Au) sputtering to impart the electrical conductivity to get top surface and back-scattered micrographs of its cross-section morphology. The ZnO-MoS_2_ photocatalyst was inherently conductive, and sample imaging was performed using a high-resolution lens at an accelerating voltage of 20 kV without gold sputtering.

#### 2.6.3. Structural Characteristics 

The surface structural characteristics of the ZnO-MoS_2_ PCM were studied by performing X-ray photoelectron spectroscopy (XPS) (VG Scientific ECSA lab Mk II system) using Al Kα X-rays (1486.6 eV) under ultra-high vacuum conditions (<5 × 10^−10^ mbar). Survey and elemental spectra were recorded while setting the analyser pass energy to 200 eV. An electron flood gun was employed for the charge compensation, and the binding energy scale of the adventitious carbon 1s core-level was referenced at 284.8 eV.

### 2.7. Studies on Photocatalytic Degradation of MB dye

#### 2.7.1. Hybrid ZnO Doped MoS_2_ Photocatalysts for MB Dye Treatment

An amount of 100 ml of 20 ppm MB solution was stirred in two different beakers with 100 mg each of ZnO and the hybrid ZnO-MoS_2_ photocatalyst. The solutions were initially left in the dark under stirring for 1 h to allow them to reach the absorption–desorption equilibrium. UV irradiation was then exposed while placing the solutions within an enclosed chamber. Sampling was done at every 15 min for hours, and collected samples were then centrifuged at 14,000 rpm for 10 min to remove any traces of the residual photocatalyst. The MB dye concentration was then determined by measuring the absorbance peak of the samples at 665 nm. The obtained data on the difference between the initial and final concentration were plotted against time to investigate the photocatalytic activity of ZnO and the hybrid ZnO-MoS_2_. 

#### 2.7.2. Photocatalysis Assisted Membrane Filtration Studies for MB Dye Treatment

**Figure 2:**
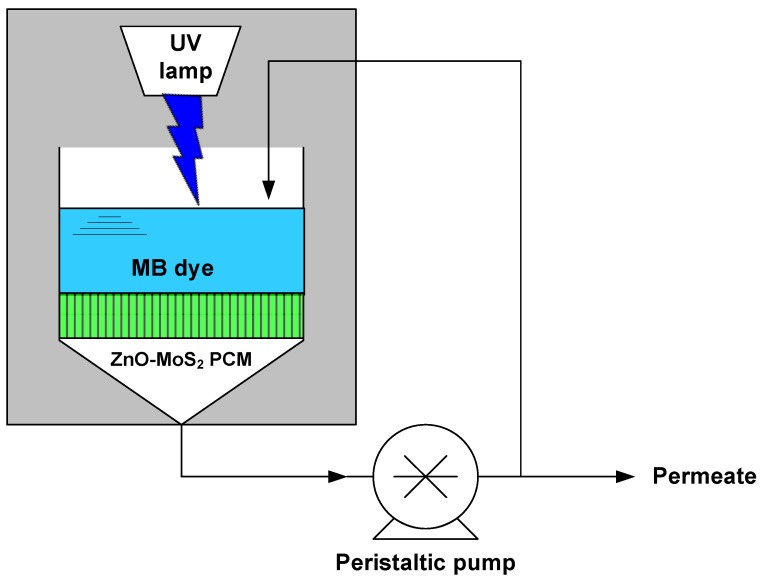
Schematic representation of UV-assisted membrane filtration set-up for the MB dye treatment.

A laboratory scale photocatalytic membrane reactor was set up, as shown in Figure 2. A UV lamp (Phillips-UVA, 9 W, RS Radionics, Dublin, Ireland) emitting a near-UV radiation of 365 nm wavelength was placed at about 3 cm above the membrane filtration set-up (Sartorius filtration apparatus, Microsart®, Göttingen, Germany) and kept together enclosed in a closed chamber as shown in Figure 2. The ZnO-MoS_2_ PCM, having a diameter of 47 mm, was mounted in the filtration set-up connected to the peristaltic pump. The feed solution containing 20 ppm concentration of MB dye was pumped into the microfiltration set-up while irradiating the ZnO-MoS_2_ PCM. Samples were collected every 30 min while recirculating the permeate back into the feed tank. All trials pertaining to the photocatalytic-assisted filtration performance evaluation and reusability of the ZnO-MoS_2_ PCM are studied using the same experimental set-up.

Initially, deionised water was passed through the virgin PVDF and ZnO-MoS_2_ PCM to compare the steady-state pure water flux. MB dye filtration studies were performed on the above-mentioned filtration set-up by employing (1) the virgin PVDF membrane, (2) the surface-modified ZnO-MoS_2_ PCM with filtration under dark (UV off) and (3) a UV-assisted filtration on the ZnO-MoS_2_ PCM. The permeate was collected to determine the flux and dye concentration to compare the efficiency and rejection ability of the virgin PVDF and modified ZnO-MoS_2_ membranes. The flux through the membranes was calculated using the following Equation (2):

(2) $$J=\frac{V}{A\times \Delta t}$$

where *J* represents the permeate flux (Lm^−2^h^−1^), *V* is the volume of the filtered water (m^3^), *A* is the active membrane area (m^2^) and Δt is the working time (h). 

The dye rejection efficiency was quantified based on the concentration of the initial feed *(C_i_)* and obtained permeate concentration *(C_p_)* using the below Equation (3):

(3) $$\mathrm{Rejection}\;\mathrm{efficiency}\;=\;\left(1-\frac{C_{p}}{C_{i}}\right)\times 100\%$$

## 3. Results and Discussion

### 3.1. Morphology of ZnO-MoS_2_ Photocatalyst

**Figure 3:**
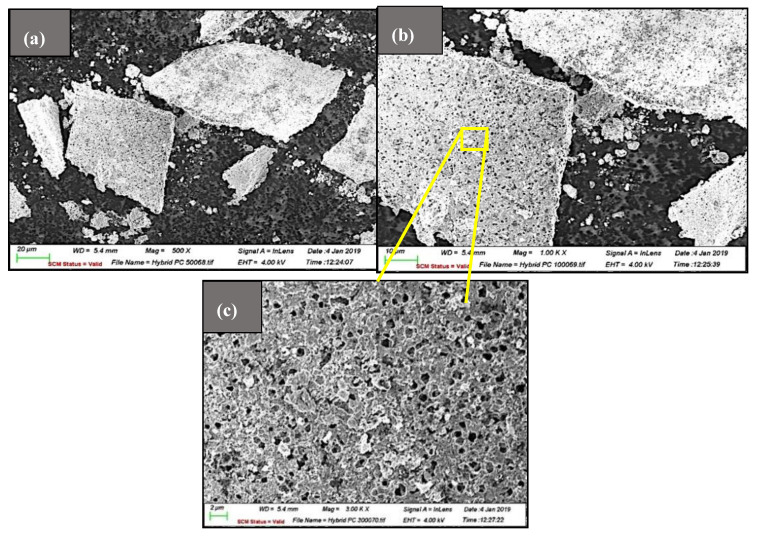
SEM images of the ZnO-MoS_2_ hybrid photocatalyst (**a**) at 500×; (**b**) at 1000×; (**c**) magnified porous surface at 3000×.

The surface morphology of the ZnO-MoS_2_ photocatalyst characterised by SEM is shown in Figure 3, wherein exfoliated few layers of MoS_2_ sheets have been decorated with ZnO on the surface. Upon exfoliation, it could be observed that the lateral size of the MoS_2_ sheets has decreased due to the sonication-induced scission. The size reduction of the lateral edges plays a significant role in rendering beneficial properties to the exfoliated 2D layers of MoS_2_, which in the present application have turned the ZnO-MoS_2_ sheets catalytically more active than the bulk. The reduction in the lateral size of ZnO-MoS_2_ is beneficial for photocatalytic applications as edge sites rather than basal planes of MoS_2_ are known to be catalytically active [29]. 

It is significant to visualise the dense surface porous structure of the MoS_2_ sheets, which would certainly offer improved permeability, owing to the higher cumulative porosity of the ZnO-MoS_2_-deposited membranes [30]. Other superior functionalities, including the catalytic activity and wettability, are expected to enhance by taking advantage of the surface pores [31]. 

### 3.2. Raman Spectral Analysis of MoS_2_ and ZnO-MoS_2_ Photocatalyst

**Figure 4:**
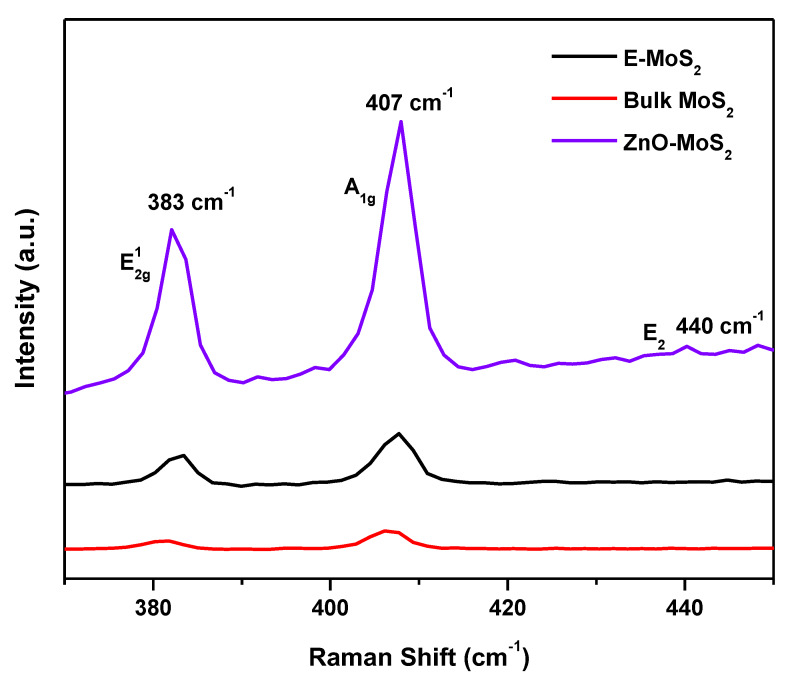
Raman spectra of the bulk MoS_2_, E-MoS_2_ and hybrid ZnO-MoS_2._

Raman spectra for the bulk and exfoliated MoS_2_ dispersion are shown in Figure 4 and the displayed two MoS_2_ peaks are the in-plane E^1^_2g_ and the out-of-plane A_1g_. Peak positions at 383 cm^−1^ for E^1^_2g_ and 407 cm^−1^ for A_1g_ confirm the presence of MoS_2_ [32,33]. The presence of a blue shift on the E^1^_2g_ peak implies that a degree of exfoliation has occurred. The absence of the A_1g_ red shift, which is typically more responsive to reducing the layer number, could be a result due to SDBS, which adhered to the basal planes of the MoS_2_ as it stabilises the dispersion through its ionic charge. When comparing the bulk and exfoliated MoS_2_, there is a blue shift from 381 to 383 cm^−1^ on the in-plane E^1^_2g_ peak which is attributed to the reduction in the number of layers [34]. 

The Raman spectra of the hybrid ZnO-MoS_2_ (Figure 4) show the characteristic peak of the A_1g_ mode specific to the exfoliated sheets of MoS_2_ which have turned remarkably sharper, attributed to the improved crystallinity due to the formation of ZnO and MoS_2_ heterojunctions. Moreover, the presence of a weaker E_2_ (H) vibrational band exhibited at wavenumber 440 cm^−1^ represents the incorporation of a hexagonal wurtzite crystal structure of ZnO in the hybrid ZnO-MoS_2_ composite [27,35]. The reduction in the Raman shift occurring at the vibrational modes of the E_2g_ and A_1g_ peaks has also confirmed the 2H-hexagonal crystal plane of MoS_2_ with a reduced number of layers [36]. However, a more plausible rationale behind the reduced shift is attributed to the extent of exfoliation, which has been successful to only a few layers rather than a monolayer exfoliation. The Raman peak positions of the few layers of MoS_2_ are known to converge on its bulk profile when the layer number ≥4, as reported [37], thus suggesting the thickness of the obtained E-MoS_2_ sample has possibly around four layers.

### 3.3. Band Gap Energy and Stability Analysis of ZnO-MoS_2_ Photocatalyst

The zeta potential of E-MoS_2_ and the ZnO-MoS_2_ hybrid was found to be −38 mV and −33 mV, respectively, thus confirming its better stability in the SDBS exfoliation medium. Indeed, a higher negative value of the ZnO-MoS_2_ hybrid relative to ZnO (−3.8mV) confirms the anchoring of MoS_2_. Further, the negative zeta potential value is attributed to the negatively charged SDBS, which rendered the ionic charge stability to the hybrid ZnO-MoS_2_ dispersion through its electrostatic repulsions between the sheets [20]. Another factor is the tendency of the surfactant to absorb at the interface of the ZnO and MoS_2_ sheets wherein the aromatic ring of SDBS attaches only to the basal plane and leaves the catalytically active edge site of MoS_2_ free [38], thereby retaining the stability and improving the photocatalytic activity of ZnO-MoS_2_.

**Figure 5:**
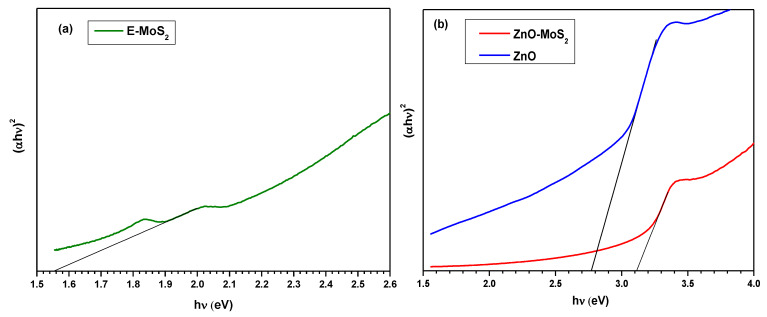
(**a**) Tauc plot for E-MoS_2,_ (**b**) Tauc plot for ZnO and ZnO-MoS_2._

The optical band gap was estimated using the Tauc plot, by extrapolating a line from the linear region on the plot of (αhν)^2^ vs. (hν), as indicated in Figure 5. The band gap of E-MoS_2_ was estimated to be ~1.55 eV; this value is in conjunction with the Raman data implying the exfoliation with most MoS_2_ sheets is achieved when lying between two and six layers. As is obtained from the energy band gap analysis shown in Figure 5b, it is confirmed that the wide band gap of ZnO ~3.2 eV [39] has been shortened to ~2.77 eV upon doping E-MoS_2_, which typically holds a narrow direct band gap energy of 1.9 eV [40,41]. The addition of the narrow indirect gap p-type E-MoS_2_ to ZnO has decreased its band gap through the creation of a p–n heterojunction [42]. The resultant energy band structure positively influenced the charge transfer of the photoinduced electrons in the conduction band of MoS_2_ to ZnO, thus leaving behind photogenerated electron–hole pairs to produce hydroxyl and superoxide free radicals [43]. The rapid generation of those free radicals eventually helped in the MB dye degradation at a rate relatively faster than employing pristine ZnO.

### 3.4. Effect of ZnO-MoS_2_ Photocatalyst Deposition on PVDF Membrane Properties

#### 3.4.1. XPS Structural Analysis

**Figure 6:**
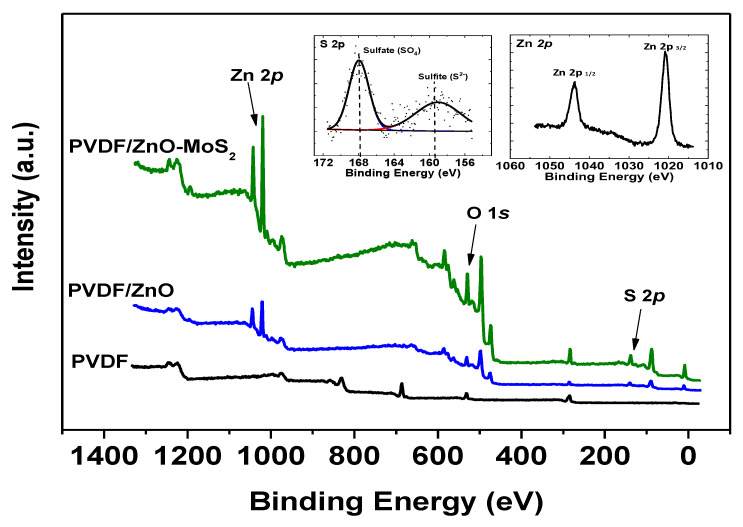
XPS binding energy spectrum of the virgin and the modified PVDF.

The chemical structural analysis of the modified PVDF surface of the ZnO-MoS_2_ PCM, as identified through XPS measurements, is shown in Figure 6. The survey scan of PVDF/MoS_2_-ZnO indicates the presence of Zn 2p, O 1s, Mo 3d, S 2p and C 1s peaks, which are compared against the virgin PVDF showing a prominent peak for F 1s. The deconvoluted S 2p binding energy peak at 160 represents the presence of sulphide and clearly demarcates it from the peak at 168 which corresponds to a sulfate molecule from the SDBS dispersion medium. The presence of the S 2p peak at the binding energy of 160 eV is indexed to the S^2−^ ions in the Mo–S bonding of disulfide in MoS_2_. Moreover, peaks at the binding energies of 1020 eV and 1045 eV are characteristic of 2p 3/2 and 2p1/2, respectively, of Zn 2p, together corresponding to ZnO [44]. It is also important to mention that there is no doublet peak corresponding to Mo-3d, confirming no existence of Mo-O bond on the surface of the photocatalytic membrane [45].

#### 3.4.2. Influence of ZnO-MoS_2_ on Morphological Characteristics 

**Figure 7:**
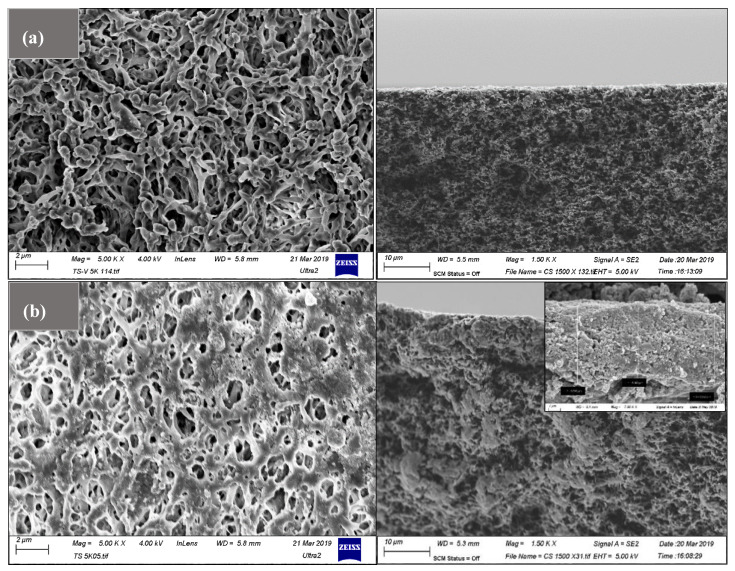
Top and cross-section morphology of (**a**) PVDF and (**b**) ZnO-MoS_2_ membrane.

The SEM image, as shown in Figure 7a, compares the virgin PVDF membrane, with its surface modified, with the ZnO-MoS_2_ photocatalyst shown in Figure 7b. 

The random restacking of the MoS_2_ layered structure enables the size reduction of the larger pores, which helps to shorten the channel through which the water solute traverses. The average height of the active ZnO-MoS_2_ was identified to be nearly ~6 microns based on the cross-section SEM imaging on the prepared ZnO-MoS_2_-modified PVDF membrane, which enabled us to infer a uniform thickness of the photocatalytic layer on the membrane surface. Hence, the orientation of a few layers of MoS_2_ has altered the pore morphology of the PVDF membrane for achieving improved filtration properties.

#### 3.4.3. Influence of ZnO-MoS_2_ on Hydrophilicity and Porosity Characteristics 

**Figure 8:**
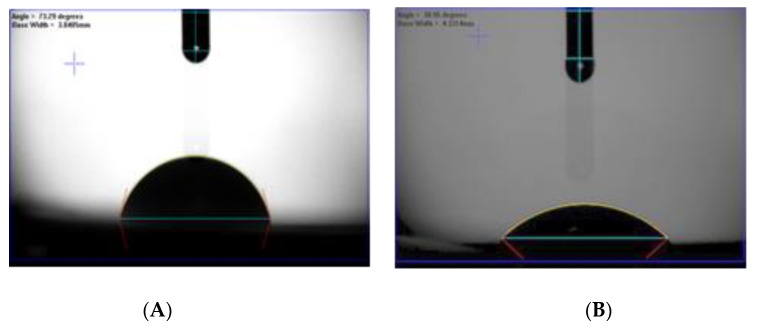
Images representing the contact angle of (**A**) the virgin PVDF and (**B**) the ZnO-MoS_2_ photocatalytic membrane (PCM).

The contact angle was recorded to investigate the wettability of the surface-modified PVDF membrane against its unmodified counterpart. The average contact angle of the modified membrane is 41.04°, which is lower than the virgin PVDF having the highest average contact angle of 72.24° due to its inherent hydrophobic nature. Images shown in Figure 8 further illustrate the higher hydrophilic membrane surface of the ZnO-MoS_2_ PCM. The significant reduction in the contact angle of the modified membrane is attributed to the hydrophilic nature of SDBS and ZnO, which has also contributed free hydroxyl groups on the surface of the ZnO-MoS_2_ PCM. 

**Figure 9:**
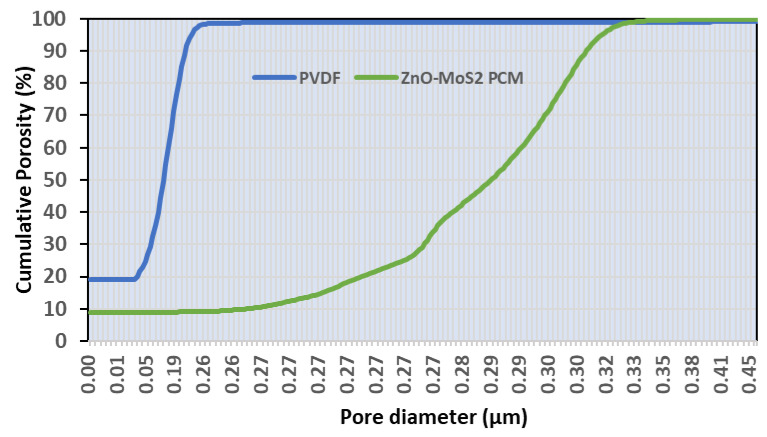
Pore diameter vs. cumulative porosity of the virgin and the modified PVDF membranes.

The mean flow pore size was also calculated to be 0.20 µm and 0.33 µm for the virgin and the modified PVDF membranes, respectively (Figure 9). In contrast to the virgin PVDF, the distribution of the cumulative porosity was across a wider pore diameter for the ZnO-MoS_2_ PCM. The improved pore volume could subsequently influence the permeation of the ZnO-MoS_2_-based PCM for filtration applications. The wet flow characteristics of the modified PVDF membrane achieving a higher porosity is due to the surface hydration of the MoS_2_ sheets, confirming the intercalation of –OH molecules. The inherent free volume of ZnO-MoS_2_ and intricate pores of the hybrid catalyst on the PVDF surface have offered enhanced porosity, as witnessed by the improved hydrophilic properties of the modified membrane. Moreover, the layered random orientation of the MoS_2_ nanosheets has also resulted in mitigating the larger pores/channels.

### 3.5. Performance Evaluation of ZnO-MoS_2_ on MB Dye Degradation

**Figure 10:**
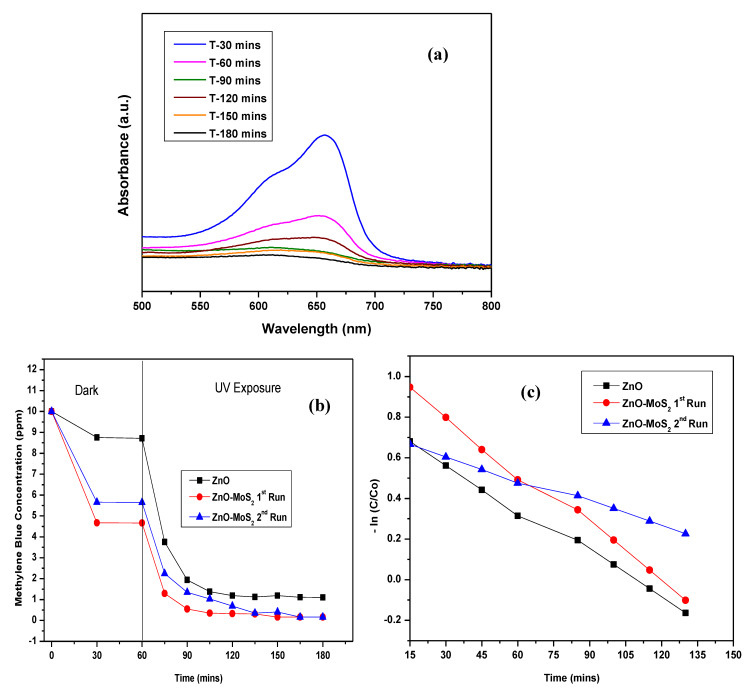
(**a**) Plot showing absorbance vs. wavelength for the MB degradation using hybrid ZnO-MoS_2_ photocatalysts. (**b**) Effect of hybrid ZnO-MoS_2_ photocatalysts on the MB dye degradation. (**c**) MB degradation kinetics for pure ZnO and ZnO-MoS_2_ across the entire irradiation time.

The photocatalytic activity of the raw ZnO-MoS_2_ powder was evaluated by the photodegradation of MB dye (10 ppm) under a long-wave 365 nm UV-A light. Based on the correlation between the absorbance recorded for the MB concentration and wavelength (~660 nm), as shown in Figure 10a, the residual MB concentration as a function of illumination time was determined and plotted, as shown in Figure 10b, to study the photocatalytic activity of pristine ZnO and ZnO-MoS_2_ for two cycles. Under a long-wave UV light, the photocatalytic efficiency of the ZnO-MoS_2_ composite is higher compared with pure ZnO with ZnO-MoS_2_ achieving 97.21% degradation and ZnO achieving 89% after 180 min, and the hybrid ZnO-MoS_2_ displayed a better initial efficiency of 87.12% compared with ZnO showing only 56.89% after 15 min, resulting from its improved adsorption of MB on to its surface. The efficient contact between the interface of the heterojunction of ZnO and MoS_2_ enables the charge separation of the electron–hole pairs owing to the diffusion of electrons in the ZnO nanoparticles to MoS_2_ at the interface [23]. The improved charge carrier density gradient and subsequent generation of an internal electrostatic field together influence the charge separation of the photogenerated electron−hole pairs in the hybrid ZnO-MoS_2_ composite, thus leading to an enhanced photocatalytic performance in the MB dye degradation. The photocatalytic degradation of MB by ZnO-MoS_2_ has been reported to follow pseudo first-order kinetics [46], governed by the rate equation $-\ln(\frac{C}{C0})=kt$ where k is the rate constant (min^−1^), and t is the illumination time in minutes. Figure 10c shows the plot of $-\ln(\frac{C}{C0})$ vs. the irradiation time from which the overall rate constants for the ZnO and ZnO-MoS_2_ degradations were found to be 0.029 min^−1^ and 0.041 min^−1,^ respectively, with the second run of ZnO-MoS_2_ showing quite a closer rate constant of 0.021 min^−1^. 

### 3.6. Performance Evaluation of ZnO-MoS_2_ Deposited Photocatalytic Membrane

#### 3.6.1. Flux Performance 

**Figure 11:**
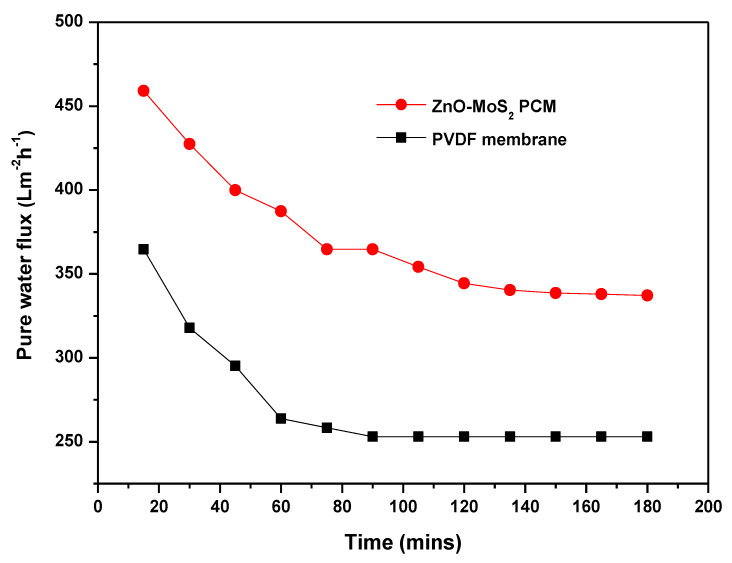
Pure water permeability of PVDF vs. the ZnO-MoS_2_ PCM.

**Figure 12:**
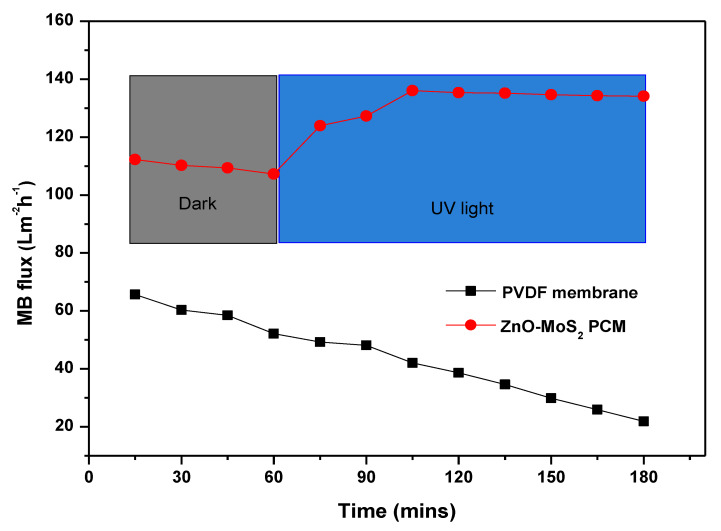
Permeability of PVDF vs. the ZnO-MoS_2_ PCM during the MB dye filtration.

In complementary to the improved hydrophilicity and porosity, the pure water permeability of the ZnO-MoS_2_ PCM has increased 25% relative to the virgin PVDF as shown in Figure 11. There has been a steady flux decline, which is evident as a result of the membrane compaction under a hydraulic pressure. It is also apparent that the interlayer spacing of the stacked MoS_2_ in its completely hydrated state has exhibited a substantial improvement of the water flux and transport nature. Similarly, the flux values from the MB dye treatment, as shown in Figure 12, indicate that the MB dye permeation from the ZnO-MoS_2_ PCM is ~70.86% higher than the virgin PVDF membrane, which is attributed to the smooth MoS_2_ nanochannels causing a lower hydraulic resistance [19]. After exposure to UV illumination at 60 min, the MB flux of the ZnO-MoS_2_ PCM has exhibited a considerable increase of about 20% at the end of the 2 h of UV exposure. This prominent effect is certainly attributed to the photocatalytic activity of the hybrid ZnO-MoS_2_ layer that eventually reduced the fouling layer resistance caused by the MB dye solutes [47]. The surface-deposited layer has offered to overcome the membrane resistance and enabled to improve the productivity while rejecting the MB feed solution. The effect of the surface properties of the ZnO-MoS_2_ PCM in overcoming the trade-off between permeability and rejection [48] is evident from the MB dye rejection performance discussed in further sections. 

#### 3.6.2. Synergistic Effect of ZnO-MoS_2_ PCM for Improved MB Dye Treatment

**Figure 13:**
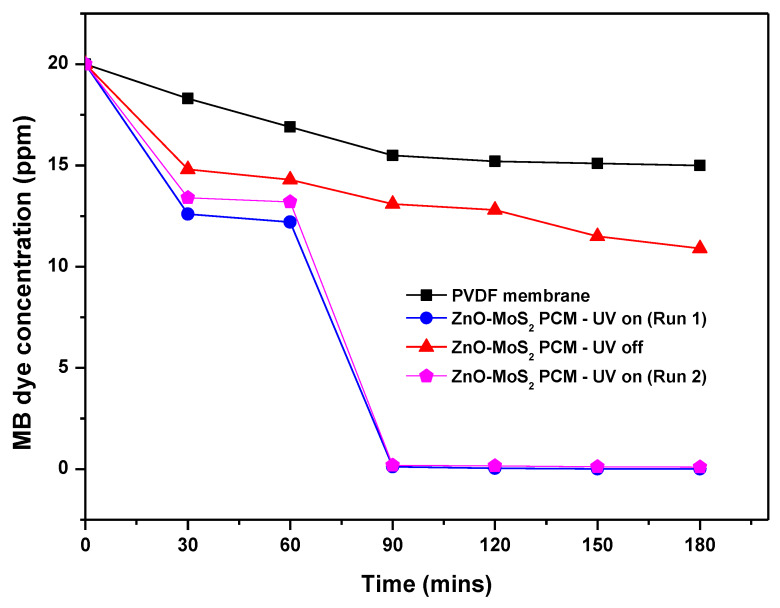
MB dye rejection studies on PVDF and the ZnO-MoS_2_ PCM.

In order to realise the synergistic effect of photocatalysis and membrane filtration performance, the as-prepared ZnO-MoS_2_ PCM was subjected to only pressure-assisted filtration under dark. After 1 h, the MB dye rejection rate of the virgin PVDF and ZnO-MoS_2_ PCM was found to be about 8.5% and 27%, respectively, thus markedly indicating no significant influence of a photocatalytic layer under dark. When exposed to UV illumination, the MB concentration reduced to about 0.12 mg/mL in 30 min with a maximum degradation of about 99.95% in 2 h (Figure 13). 

This clearly shows a significant improvement in the MB rejection performance of the ZnO-MoS_2_ PCM and evidences the pronounced synergism of photocatalysis and filtration. The catalytically active edges of MoS_2_ induced the photocatalytic production of radicals which bind to the pollutants, thus resulting in a complete mineralisation [29]. The enhanced photocatalytic performance of PVDF/MoS_2_-ZnO is attributed to the catalytically active site of MoS_2_ wherein stacking few layers of the hybrid ZnO-doped MoS_2_ sheets [21] on the PVDF membrane substrate has offered advantageous effects on improving the photocatalytic-assisted dye filtration when compared with a randomly packed or reaggregated ZnO-MoS_2_ dispersion suspended in an MB dye solution. Further, the influence of surface-selective pore characteristics has also been evident from the extent of the rejection rate of about 45.5% achieved by the ZnO-MoS_2_ PCM membrane under no UV irradiation, as against the virgin PVDF membrane exhibiting only a 25% rejection. 

#### 3.6.3. Reusability of ZnO-MoS_2_ PCM

**Figure 14:**
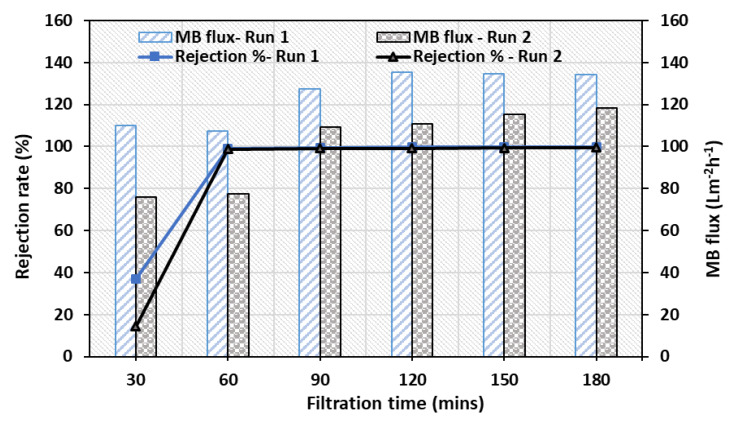
Permeation and rejection efficiency of the ZnO-MoS_2_ PCM for two subsequent filtration cycles.

In order to evaluate the reusability of the ZnO-MoS_2_ PCM, a UV-assisted membrane filtration was performed on the same membrane back washed with water for 10 min and fed with the fresh MB dye solution. The performance, as measured by the permeate flux during the second run, has shown a decrease of about 30% compared with the initial run. The reduction in MB flux rate to about 118 Lm^−2^h^−1^ has been observed during the second run as against 134 Lm^−2^h^−1^ obtained for the initial filtration run. However, the rejection rate of the reused ZnO-MoS_2_ PCM has only reduced marginally (~1%), thus demonstrating the stability and restoration of the photocatalytic efficiency of the ZnO-MoS_2_ layer (Figure 14), reaching nearly a 99.45% removal of MB dye. The reusability of the prepared ZnO-MoS_2_ PCM is much faster, unlike using the hybrid ZnO-MoS_2_ in powder form, where additional steps like centrifugation and drying are required to recover the photocatalyst [49]. This confirms that the reusability of the ZnO-MoS_2_ PCM is quite facile and efficient upon a comparison with the hybrid ZnO-MoS_2_ photocatalyst in powder form. 

## 4. Conclusions

This photocatalytic membrane fabrication by depositing the exfoliated dispersion of the hybrid ZnO-MoS_2_ photocatalyst implicates the proof of the concept of utilising the highly stable organic polymeric substrate with a simultaneous catalytic activity for an overall improvement of a water/wastewater treatment. The influence of catalyst immobilisation on the PVDF membrane substrate has offered superior surface functional properties. Photocatalytic degradation studies have also proved that the heterogeneously structured ZnO-MoS_2_ nanocomposite is photocatalytically more efficient than pristine ZnO. Robust advances in membrane surface modification strategies enable to utilise the multi-functional properties of MoS_2_ without altering its desired molecular channels to eventually result in breakthrough advances in the field of membrane separation processes. The current work also brings scope for research studies on precisely tuning the physico-chemical properties of surface-modified photocatalytic membranes to design scalable and continuous photocatalytic membrane reactors.
